# Understanding Technology, Changing the World

**DOI:** 10.1007/s11569-021-00410-x

**Published:** 2022-01-05

**Authors:** Christopher Coenen

**Affiliations:** Institute for Technology Assessment and Systems Analysis (ITAS), Karlsruhe Institute of Technology (KIT), Karlsruhe, Germany

Largely due to the continuing difficulties caused by and connected with the pandemic, there was no time to write editorials for this year’s two earlier issues of our journal *NanoEthics: Studies of New and Emerging Technologies*. The present editorial for the third issue thus constitutes a kind of review of 2021.

I was particularly happy that the first issue of the year included a special section on ‘emancipatory technology studies’, a topic close to my heart. It was guest-edited by *Philipp Frey, Simon Schaupp and Klara-Aylin Wenten* who point out in the section’s introduction that “against the backdrop of the advent of techno-utopianism in both libertarian and conservative forms and technologically mediated attacks on privacy and labour standards, critical scientists have often resorted to […] criticising technological progress altogether”. They argue that this “urge is more than understandable”, but that “it can reinforce the risk of reifying technology as the driving force behind problematic societal developments, rather than regarding it as the product of a politically contested field of socio-material practices”. Their aim is to help create a kind of new field called ‘emancipatory technology studies’ (ETS) that combines traditions of critical theory, science and technology studies (STS) and critical sociology of work. In their introduction, which is well worth reading, the guest editors discuss why such a new field is needed nowadays, pointing to automation and other challenging and pressing topics for research. Furthermore, they explain that the common denominator of emancipatory politics is that it is “dedicated to dismantling societal power relations”, or to helping, as Karl Marx famously put it, to overthrow all relations in which Man is a debased, enslaved, abandoned, despicable being. One goal of ETS defined by the guest editors is to “open up technology design and use to democratic negotiation”. In their view, one should avoid joining “the voices blaming (technological) rationality itself for the perennial misery of human history” – the ‘demon technology’ (*Dämon Technik*) of (not only) conservative and some fascist German philosophers – and “hypostatising technological development” by “separating it from the purposes it serves and its concrete embeddedness in social relations”. ETS should not contemplate the form of technological development idealistically but should rather understand technology as being closely linked to societal conditions. In this context, they discuss the thoughts of Theodor W. Adorno, arguing that his works should not be perceived as belonging to *Dämon Technik* literature. As Frey and his colleagues emphasise, ETS not only strive to develop a theory of technology in society but also to involve emancipatory practices of research. They believe that this includes democratising the relationship between researcher and research subject in the production of knowledge – and it is not only this that shows that ETS can also align with concepts and practices of responsible research and innovation (RRI), including the rise of citizen science activities and their funding, which have been developed since the beginning of the current century. In contrast to other RRI research and activities, however, ETS, as the guest editors point out, should particularly build on and continue the tradition of ‘action research’ and conduct “investigations together with those at the bottom of current societal hierarchies”.

The very fine articles in the special section guest-edited by Frey, Schaupp and Wenten are testament to the potential such ETS Offer when it comes to understanding technology and helping to change a world that, ever since the collapse of the Soviet Union, has experienced a turbulent period of capitalist global domination and increased digitalisation of societies and work life. I will not discuss these articles in great detail, because they have already been introduced by the guest editors, but a few remarks are warranted: *Georg Jochum* has contributed a rich theoretical and historical analysis to the special section and, in the light of this analysis, argues for a posthumanist emancipation project. He points to various "dialectics of emancipation", for example that bourgeois emancipation meant liberation from traditional rule and at the same time implied the freedom to exercise power over others, that the national emancipation movements of the nineteenth century culminated in the fascist catastrophe, that the emancipation of the bourgeois-capitalist subject also implied the right to subordinate other people as wage-dependent workers to the power of capital, and that the emancipation of the individual led to the elimination or marginalisation of concepts of a community-oriented organisation of work and life. From Francis Bacon to Elon Musk and from Max Horkheimer to current critical posthumanists, Jochum discusses a wide range of thinkers and positions, including a critique of transhumanism, and ends with a plea for a critical posthumanism on which ETS could be based. The other four articles in this special section are also empirically rich: Johan Söderberg and Maxigas provide us with an insightful analysis of hacker practices and spaces and distinguish hackers from adjacent movements such as citizen scientists or makers. They propose a conceptual framework for analysing the relationship between social emancipation and alternative technology development, arguing that the key is the “functional autonomy” of the collective of technology users and developers vis-à-vis the state and capital. Based on previous empirical work on three hacker projects, Söderberg and Maxigas emphasise that this functional autonomy of hackers rests on three pillars: technical skills, shared values and collective memory. In their view, the odds are still stacked against hackers in capitalism even when all three pillars of autonomy are firmly in place, but – and this is a central point of the authors – these favourable conditions for resisting capital are rarely present in such looser constellations as citizen science and makerspaces. *Alev Coban and Klara-Aylin Wenten* analyse the fashionable concept of ‘agile work’, arguing that it often comes with strong assumptions, as it is seen as an inevitable tool that can be universally integrated into different workplaces and has the same outcome everywhere in terms of flexibility, transparency and flat hierarchies. They challenge these assumptions by conceiving of agile work as a ‘matter of care’ and applying this feminist concept to technology development. The authors analyse the power dynamics underlying the invisibilisation of care work and argue for ETS to make visible those socio-technical practices that are going on in the background and to reveal hierarchies between, for example, valued and non-valued work or responsibilities in technology development. In his article, *Simon Schaupp* develops a multi-level framework for analysing bottom-up politics of technology at the workplace. It draws on a multi-case study on algorithmic management of manual labor in manufacturing and delivery platforms in Germany and argues for a concept of ‘technopolitics’ which refers to three different arenas of negotiation: (1) the arena of regulation, where institutional framings of technologies in production are negotiated, typically between state actors, employers’ associations, and unions; (2) the arena of implementation, where strategies of technology deployment are negotiated; and (3) the arena of appropriation, in which different organisational technocultures offer contesting schemes for the actual use of technology at work.

As Schaupp’s contribution focuses on worker agency as “technopolitics from below” and shows how workers can influence the concrete outcomes of digitalisation projects, it can be read very profitably together with the article by *Jamie Woodcock* on the perspectives of labour and resistance in digital capitalism, in which the latter combines a Marxist analysis emphasising human action vis-à-vis technology with an empirical approach in the (originally Italian) tradition of workerism. Critiquing actor-network theory as well as academic post-operaism, Woodcock emphasises the contemporary relevance of early Italian workerism of the 1960s and 1970s, in which the focus on the dynamics of workers' struggle evolved into an emphasis on workers' autonomy vis-à-vis capital – and also the importance of refusing to work. Key to this approach is the practice of ‘worker inquiry’, which involves a different approach to understanding platform work than much of STS, whose focus is on digital technologies. Rather than focusing on the algorithm, the platform or other parts of the technology, it takes, as Woodcock writes, the experience of workers as his starting point. His study draws on research that began in 2016 before the first strike by Deliveroo riders in London. As is part of the methodology, this involved both research and organizing: seeking to understand the work with drivers in order to find new ways of organising. In his Marxist analysis, Woodcock argues that the use of automation was long ago an attempt to increase control in the workplace and extend capitalist planning from the factory to the market and then to wider society. Technological automation has become a threat to autonomy by depriving workers of the opportunities and spaces to resist capitalist control; and these experiences are increasingly used outside the workplace. Automation is at the same time an attempt by capitalists to emancipate themselves from the working classes – a tendency that is evident today in the pronouncements of the tycoons of digital capitalism like Jeff Bezos. The emancipatory potentials of technology are thus counteracted by its class nature. Woodcock's article ends with a plea to see new technologies both as an arena for struggle and as new possibilities for monitoring, surveillance and oppression, and to help express the emancipatory potential of technology through processes of workers' inquiry and joint research with workers.

The special section guest-edited by Frey, Schaupp and Wenten may remind us that the liberal view of the world – which has strongly shaped the field of applied ethics – should not be mistaken for the world itself, whose current features are not a “natural” state of social affairs that can be taken for granted but a capitalist order that liberalism is rarely able to understand.

The fact that thought traditions such as Marxism and anti-capitalist feminism are marginalised in academia for ideological reasons is an anomaly that is currently ending. This is due on the one hand to the undiminished relevance of ideas stemming from these traditions in recent and current theories and practices in STS, which also make it appear to be appropriate to revisit the classics of dialectic philosophy [1]. On the other hand, Marxist and other emancipatory traditions, such as anarchism, anti-imperialism or postcolonial critique, are well-suited to helping us analyse and practically tackle a problem that plays a key role – or even *the* key role – in understanding technology in global society: the crass discrepancy between the highly advanced state of the productive forces in many parts of the world, including huge technological potentials everywhere, and the deplorable state of affairs faced not only by the poor in less industrialised regions but also by the many people in so-called ‘rich countries’ who have to work in essential but usually hard and low-paid jobs or in often fairly well-paid but meaningless ‘bullshit jobs’ [2]. As I wrote in the editorial of the second issue of *NanoEthics* in 2017, we need to ask how our societies can be re-organised in light of the rapid progress that is taking place in many technological fields and what we all going to do once we reach the point at which the Western utopia – of humankind being freed from toil and all kinds of unpleasant labour – becomes a reality, at least in Europe and North America. Bertrand Russell, in his essay *In Praise of Idleness* (1932), argued that this has already been achieved in principle and that only an outdated ethic and a wrong distribution of labour prevent its full realisation [3]. Although in my opinion this is not yet true for large parts of the world, which are not even near to any vision of automation and are still waiting for their full integration into capitalism—albeit with considerable political and social resistance—, he was right about these two main reasons preventing a better future for humanity. Against this backdrop, and with a view to making our technosphere truly and sustainably habitable, it is one of the most distinguished tasks of philosophy to again develop an integrative notion of socio-technical progress [4].

The old quip that it is harder today to imagine the end of capitalism than the end of the world may still be true, but the highly repetitive dystopias created by the culture industry these days are often nothing but tedious. New practices of a sharing economy as well as the surge of discussions about and first experiments with universal basic income (UBI) programmes may be signs that our consciousness is beginning finally to grasp the realities. The pro-capitalist ideological fog produced in an increasingly desperate and shameless manner by political demagogues and by the mass media, including the so-called alternative media on the extreme political Right that resort to ever more irrational conspiracy thinking, is often being dispersed.

Trends such as the increasing fascination with the idea of a UBI have also created a new context for ethical reflection, and this includes a growing awareness that in digital capitalism – the brand that actually exists rather than the visions developed in pro-capitalist ideological projects –, work violates human autonomy and dignity beyond the sweatshops, mines, factories and brothels in the most exploited areas in the global South. Digitalisation is also radically transforming white-collar work in many areas, giving rise to what we might term a new bureaucrapitalism that combines ever greater control of employees with an uncontrolled proliferation of bureaucracies that are increasingly used to organise meaningless competition between employees or units within one institution and within publicly funded systems such as the science system.

The second *NanoEthics* issue in 2021 included another special section on a topic that is pretty much at the centre of our journal’s thematic scope: the question of hype, that is to say excessive future promises and expectations, in discourse on new and emerging technoscientific fields, and on biotechnology, nanotechnology and artificial intelligence (AI) in particular. In their introduction, guest editors *Franz Seifert and Camilo Fautz* argue that nanotechnology is distinctive in two ways. First, the technology was surrounded by hype from the beginning, justifying funding programmes and driving financial, scientific and innovative momentum, followed by slumps of disillusionment and disengagement after promises and expectations were not met. Secondly, no other technology had previously received so much attention from the social sciences and humanities at an early stage. Seifert and Fautz present bibliometric data showing that the hype surrounding nanotechnology, including the hype in the social sciences and humanities, is long a thing of the past. With the special section, which contains few but diverse contributions, the guest editors aim to contribute to the study of the increasing involvement and integration of social sciences and humanities in science, technology and innovation policy. They argue for a critical self-assessment and self-reflection of the involved social scientists and scholars as well as for conceptual approaches that critically question the role of these fields in science, technology and innovation policy. In his contribution to the special section, *Frederick Klaessig* provides us with an insightful comparison of biotechnology, nanotechnology and AI. He argues that that the promissory claim for nanotechnology to exploit unique phenomena challenged regulatory practice, while biotechnology claims were often the fruit of themes found in the shared educational experience of biotechnology researchers and regulators. Based on a comparison of developments in these two fields, Klaessig develops a prospective AI trajectory using the example of driverless cars. *Jantien Schuijer, Jacqueline Broerse and Frank Kupper* focus in their article on the challenges faced by social scientists and humanities scholars in engaging the public in new scientific and technological developments. Their contribution is exactly the kind of critical self-reflection by such social scientists and scholars that was called for by Seifert and Fautz. Drawing on their own experience in an EU-funded project on responsible nanotechnology, they define five different roles for social scientists and humanists in such engagement projects: the ‘engaged academic’, the ‘deliberative practitioner’, the ‘change agent’, the ‘dialogue capacity builder’ and the ‘project worker’. The authors question the assumption that practical experience and a reflexive attitude will foster the integration of these different roles over time, and argue that more needs to be done to ensure the process qualities of both public engagement practice and academic work in the field. They believe that educational programmes and training opportunities are needed for social scientists and humanities scholars who want to integrate a variety of roles into their work on public engagement with science, technology and their governance. Last but not least, role integration would also need to be institutionally valued and supported to increase its chances of societal impact. Schuijer and colleagues warn that as long as the impact of social scientists and humanities scholars involved in such activities is assessed only in terms of academic publications, their ability to contribute to real change will remain marginal. Asking them to work on socio-technical integration within structures that remain disconnected from actual influence on research and innovation policy, they argue, hinders the realisation of the systemic changes discursively promoted at EU level.

The special section guest-edited by Seifert and Fautz was rounded off by a highlight of self-reflective scholarship that also focuses on public engagement: an invited contribution by *Bernadette Bensaude-Vincent* on French public dialogues on nanotechnology. The author first reports from an insider's perspective on the public debates organised by the civil society group VivAgora in the context of the fierce national controversies on nanotechnology (2005–2009). She then describes the permanent forum NanoRESP, which was opened in 2013 when the controversies in this field subsided. Based on this case study, Bensaude-Vincent argues that the STS ideal of science-society co-production gradually gave way to a more modest co-learning process between stakeholders in the 2010s. Her contribution is a fascinating account of nanotechnology discourse in the country where it became most heated, involving massive political conflicts in and about official public engagement activities, militant protests against nanotechnology, and clashes of worldviews. Bensaude-Vincent agrees with Seifert and Fautz – and in all likelihood with all of us who have followed, taken part in or studied the developments since the turn of the millennium – that the nano hype is now well and truly over.

What is also obvious, however – not least from the contents of our journal – is that social science and humanities research on nanotechnology has matured and is often able to make important contributions not only to better understanding and helping improve the field itself but also more generally science, technology and their governance.

This year, this has been the case for example with several other articles in the second issue. When *Maurice Edward Brennan and Eugenia Valsami-Jones* analyse in their discussion note the ‘Safe by Design’ approach developed for nanomaterials as a template for a new sustainable innovation approach for advanced materials, they do this against the backdrop of increasing global concerns regarding sustainability. This approach requires assessment of potential toxicity risks earlier in the innovation cycle (i.e. concurrently with chemical functionality and potential commercial applications), offers future options for reducing animal testing, promotes a culture of shared responsibility for ethical and sustainable outcomes in the innovation process by encouraging early dialogue between groups with vested interests, and offers the prospect of a more democratic innovation process by involving civil society actors in decisions about product safety, commercial applications and social benefits. According to the authors, these four features, taken together, offer the prospect of a new social contract between science, technology and society in terms of sustainable innovation in advanced materials – and a model for other evolving technologies to adopt when striving for a toxic-free environment.

Furthermore, social-scientific and philosophical research on nanotechnology has become less centred on North America and Northern or Western Europe and culturally more diverse in general. In 2021, two *NanoEthics* articles in particular were evidence of this, both of which likewise featured in the second issue: *Şeyma Çalık, Ayşe Koç and Oktay Aslan* carried out a content analysis of news about nanotechnology published in Turkish newspapers in the 2010s. One of their findings was that the analysed mass media discourse on nanotechnology in Turkey, though fairly positive, is restricted for the most part to the local news sections of the newspapers. The authors also concluded that the issue of the risks of nanoscience and nanotechnology is not addressed sufficiently in Turkish media. Another example of increased cultural diversity of social-scientific or philosophical studies on nanotechnology is *Maciej Jarota*’s analysis of EU regulations concerning health and safety at work with nanomaterials, in which he discusses this topic within the context of the Catholic vision of human work. Based on his sympathetic analysis of this vision, Jarota argues for more specific European regulations while at the same time urging employers to proactively develop their own occupational health and safety (OH&S) policies for those who work with nanomaterials.

The current third issue of *NanoEthics* in 2021 also demonstrates the broad thematic scope of social-scientific and humanities research on nanotechnology: *Irini Furxhi, Finbarr Murphy, Craig Poland, Martin Cunneen and Martin Mullins* present the results of a case study of nano‑enabled textiles production and use them to reflect on the precautionary principle. The authors therefore combine a detailed risk analysis and assessment with a discussion of the precautionary principle in this context, arguing that the application of the principle, though often appropriate, should not prevent a more general risk governance process. *Adam Kokotovich, Jennifer Kuzma, Christopher Cummings and Khara Grieger* have carried out a study in which they took a closer look at how nano-agrifood researchers understand responsible innovation (RI). To this end, they conducted 20 semi-structured interviews with such researchers from industry and academia in the United States, asking them to describe their RI definitions, practices and motivations. Among other things, they found that the purpose of RI is seen by researchers as a means to protect their reputation and avoid litigation, but also as a means to improve human well-being and solve societal problems. In the view of Kokotovich and colleagues, this tension shows how important it is to pay careful attention to the way in which RI is envisioned, as the desire to “engage stakeholders” can be for very different ends. However, this does not mean that all applications of RI that are in alignment with the private sector should be questioned, as such alignments can, in the authors’ words, “open the space to explore how private sector innovation can pursue a broader set of ends than just economic profit”. On the other hand, the authors warn that if RI is widely envisioned (even in government and academic public institutions) as aligning in an unproblematic manner with the interests of private industry, this may indicate an imbalance such as “a trend of marketization and academic capitalism”.

Last but not least, studies on nanotechnology are still making major contributions, including in terms of methodology in particular, to the growing field of research on public engagement with new and emerging science and technology and their governance.

One fine example of this is an article by *Sikke Jansma, Anne Dijkstra and Menno de Jong*, which is also included in the current issue. It looks at citizen engagement in the development of nanotechnology for health, firstly by examining the aspects of these technologies on which citizens are able to make suggestions, and secondly by examining the values on which their suggestions are based. The study is based on eight focus groups, each lasting about 6.5 h and involving a total of fifty Dutch citizens. The citizens made suggestions on various technological aspects, focusing mainly on the implementation and use of technologies. Their suggestions often stemmed from concerns about potential effects of the technologies and were mainly based on the values of wellbeing, autonomy and privacy. As one of the generalisable findings of their study, Jansma and her colleagues emphasise the importance of analogies with existing technologies as a way for citizens to make sense of new and emerging technologies and to formulate their arguments and suggestions. Another fine example is a methodologically advanced study, once again courtesy of *Jantien Schuijer, Jacqueline Broerse and Frank Kupper,* that they contributed to the first issue in 2021. In the article, the authors aim to explore how situated speculative prototyping by citizens – which they nicely call ‘citizen science fiction’ – can help them to contextualise new and emerging technologies such as nanotechnology. By ‘contextualisation’ they mean the process by which citizens make abstract technological notions more tangible and embedded in the social and cultural textures of our (future) societies, in order to make sense of ambiguous, uncertain, and complex technological futures from their own experience and perspective. Using empirical data from five NANO2ALL citizen dialogues across Europe, they reflect on the potential advantages that participatory design fiction could offer in the context of public engagement and RRI, though they also highlight some of its pitfalls that – in their view – have received too little attention in the literature to date.

The relevance of RRI and the maturing of this area of research is testified by the *International Handbook on Responsible Innovation – a Global Resource* (2019) [5], edited by René von Schomberg and Jonathan Hankins. I am very happy that *Steffi Friedrichs* has provided us with a wonderful review of this handbook, published in the second issue of 2021. I wholeheartedly recommend that you read this review, which introduces and discusses the handbook against the background of both the COVID-19 pandemic and the history of responsible innovation efforts since the 2000s.

The notion of responsibility is also the focus of an article written by *Sergio Urueña* for the current issue of *NanoEthics*. In it, the author offers a fascinating in-depth analysis of theoretical discussions on anticipation in STS and technology assessment (TA), including important contributions in that journal, such as the highly influential critique of speculative ethics put forward by Alfred Nordmann in the very first issue of *NanoEthics*. Urueña's aim is to show that Nordmann's critique of speculative ethics and his later critique of certain STS and TA approaches can be made fruitful, even if one disagrees with some aspects of that critique. His article is a defense of anticipatory STS and TA and, quite in line with Nordmann and others in the field, for a critical hermeneutic approach.

As someone who often works on neurotechnologies, I am happy that we have often been able to include in *NanoEthics* important articles on ethical and other philosophical, societal and cultural aspects of these truly fascinating technologies. In the current issue, we have two articles dealing with them: *Jennifer Schmid, Orsolya Friedrich, Stefanie Kessner and Ralf Jox* present the results of a survey about brain‑computer interfaces (BCIs) that was conducted in Germany. One of its findings was the strong support for the view that the individuals using BCIs are responsible for the BCI-mediated actions and for the underlying view that these actions still count as human actions. Because BCI users themselves – as the authors found in another study – apparently tend to deny their responsibility, at least for unsuccessful or harmful actions, Schmid and her colleagues argue that coherent rules should be developed to ascribe individual agency and responsibility in the context of BCI use. Last but not least, *Benedict Charles Taylor‑Green* has provided us with the only *NanoEthics* art-science interaction article in 2021 (though more will follow soon in 2022). I love his ekphratic piece on neurotechnologies and hope you will enjoy it too in the new year, for which I wish you all the best, expressing my hope that humanity will be able to overcome the pandemic in 2022 and win the fight against the virus, everywhere.
